# Systemic Antibiotic Prophylaxis in Maxillofacial Trauma: A Scoping Review and Critical Appraisal

**DOI:** 10.3390/antibiotics11040483

**Published:** 2022-04-05

**Authors:** Femke Goormans, Ruxandra Coropciuc, Maximilien Vercruysse, Isabel Spriet, Robin Willaert, Constantinus Politis

**Affiliations:** 1 Department of Oral and Maxillofacial Surgery, Faculty of Medicine KU Leuven, University Hospitals Leuven, Campus Sint-Rafaël, Kapucijnenvoer 33, 3000 Leuven, Belgium; ruxandra.coropciuc@uzleuven.be (R.C.); maximilien.vercruysse@uzleuven.be (M.V.); robin.willaert@uzleuven.be (R.W.); constantinus.politis@uzleuven.be (C.P.); 2 OMFS-IMPATH Research Group, Department of Imaging and Pathology, Faculty of Medicine KU Leuven, University Hospitals Leuven, Campus Sint-Rafaël, Kapucijnenvoer 33, 3000 Leuven, Belgium; 3 Department of Pharmaceutical and Pharmacological Sciences, Clinical Pharmacology and Pharmacotherapy, Faculty of Medicine KU Leuven, Campus Gasthuisberg, Herestraat 49, 3000 Leuven, Belgium; isabel.spriet@uzleuven.be; 4 Pharmacy Department, University Hospitals Leuven, Campus Gasthuisberg, Herestraat 49, 3000 Leuven, Belgium

**Keywords:** antibiotic prophylaxis, anti-bacterial agents, maxillofacial injuries, bone fractures, infections

## Abstract

Infection after maxillofacial trauma remains an important complication, with a significant socio-economic impact. While consensus exists that systemic antibiotic prophylaxis reduces the risk of infection in the management of maxillofacial fractures, the type, and duration remain controversial. Therefore, the purpose of this scoping review was to provide an overview of the current evidence that supports the use of prophylactic antibiotics in the treatment of maxillofacial fractures. A comprehensive literature search on 1 January 2022, in PubMed, Web of Science, Embase, and Cochrane, revealed 16 articles. Most studies focused on the duration of systemic antibiotic prophylaxis and compared a one-day to a five-day regimen. Included studies showed considerable variability in design and research aims, which rendered them difficult to compare. Furthermore, a variety of antibiotic regimens were used, and most studies had a short follow-up period and unclear outcome parameters. This scoping review demonstrates the lack of well-constructed studies investigating the type and duration of systemic antibiotic prophylaxis in the treatment of maxillofacial trauma. Based on the included articles, prolonging antibiotic prophylaxis over 24 h for surgically treated fractures does not appear to be beneficial. Furthermore, there is no evidence for its use in conservatively treated fractures. These results should be interpreted with caution since all included studies had limitations.

## 1. Introduction

Infection after maxillofacial trauma remains an important complication [1], leading to significant morbidity and increased healthcare costs [2]. Overall, infection rates after maxillofacial fractures vary widely across studies and range from 0% to 62% [3,4]. Accurately estimating the incidence and impact of this complication is hampered by the lack of a clear definition, and the variability in outcome parameters renders existing studies difficult to evaluate and compare [5].

Systemic antibiotic prophylaxis is an accepted strategy to prevent infection in daily clinical practice [6]. However, the optimal type and duration remain controversial [7]. An awareness of the need for standardized, evidence-based guidelines has increased in recent years [8]. Furthermore, antimicrobial resistance (AMR) is increasing, and physicians are becoming aware of the importance of limiting antibiotic use [9].

Contemporary guidelines state that antibiotic prophylaxis should be provided for no longer than 24 h [10]. However, these recommendations are mainly based on a handful of clinical studies and uncertainty persists regarding the optimal antibiotic regimen for infection prevention. Previously published systematic reviews often included studies with a significant risk of bias, leading to a misrepresentation of the currently available evidence [1,3,6,7,11,12,13,14,15,16,17]. For these reasons, and on a worldwide scale, heterogeneity in infection prevention protocols still exist. Recent international surveys among maxillofacial trauma surgeons indeed concluded that most surgeons continue antibiotic prophylaxis longer than proposed, which leads to an important overuse of antibiotics [16,17]. Similar issues are currently encountered in infection prevention protocols for long-bone fractures [18,19].

The purpose of this scoping review was to investigate the association between the type and duration of systemic antibiotic prophylaxis and infection in the treatment of maxillofacial fractures. Furthermore, this study provides an overview of the currently available evidence with respect to this topic. Moreover, the aim is to identify the bottlenecks that prevent the maxillofacial community from adhering to guidelines and illustrate the implications of antibiotic overuse.

## 2. Methods

All aspects of the five-steps methodology proposed by the Joanna Briggs Institute (JBI) guidance were followed and the study was written according to the Preferred Reporting Items for Systematic Reviews and the Meta-Analyses extension for Scoping Reviews (PRISMA-Scr) guidelines [20].

### 2.1. Data Sources and Search Strategy

The search strategy was peer-reviewed by a certified librarian. A comprehensive search was performed on 1 January 2022, in the PubMed, Web of Science, Embase, and Cochrane databases. The main search concepts were maxillofacial trauma and antibiotic prophylaxis. No date restrictions were applied. Search strings for each database are provided as Supplementary Data.

### 2.2. Inclusion/Exclusion Criteria

To be included, published studies were required to present original data with the primary aim to investigate the association between systemic antibiotic prophylaxis (i.e., type, duration) and infection, in both surgically and non-surgically treated patients with maxillofacial fractures. There were no restrictions related to the type or timing of the surgical intervention or the type or class of antibiotics. Furthermore, studies were only included when a comparison group was provided and when the antibiotic prophylaxis duration was clearly described. Moreover, studies needed to clearly describe which fractures were treated surgically and which were treated non-surgically. Exclusion criteria were articles that focused on basilar skull fractures and non-traumatic fractures (i.e., pathological fractures). In addition, case reports, published abstracts, conference posters, letters, articles in languages other than English, and articles of which the full text was not available, were excluded.

### 2.3. Study Selection

**Figure 1:**
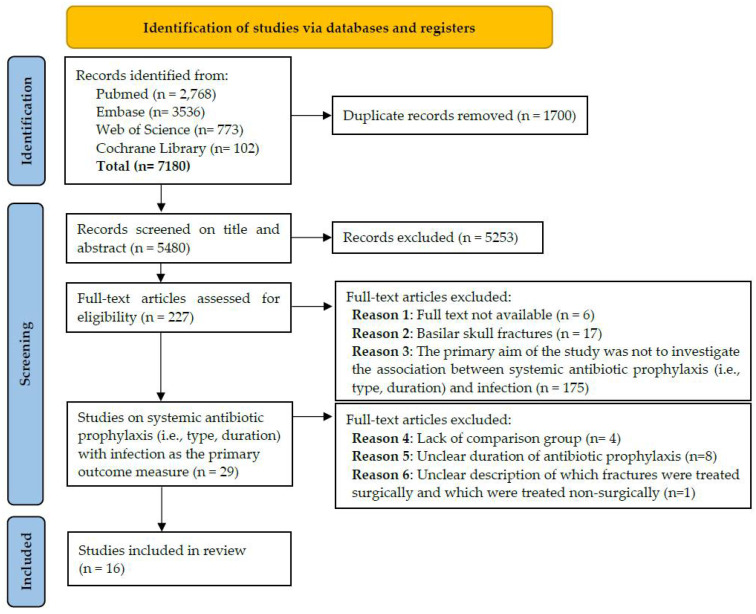
PRISMA 2020 flow diagram [21].

References were collected in EndNote and duplicates were removed. Two authors (FG and MV) independently screened the titles and abstracts of references identified in the search. In case of disagreement, a third reviewer (RC) was available for an additional discussion. After the selection of the title and abstract, potentially relevant articles were included for full-text screening. A PRISMA flow diagram provides an overview of the selection process and the number of papers retrieved and excluded (Figure 1).

### 2.4. Data Extraction and Assessment of Evidence Level

Two authors (FG and MV) independently extracted data from the selected studies and recorded the data for each included study in the ‘characteristics of included studies’ tables (Table 1 and Table 2). An assessment for possible bias was performed using the ROBINS-I tool for non-randomized studies [22] and the RoB 2 tool for randomized controlled trials [23].

## 3. Results

Overall, 7180 references were collected in EndNote. After the exclusion of duplicates, we retrieved 5480 articles. Based on the title and abstract, 5253 articles were excluded. Of the remaining 227 articles, 16 were included in this scoping review (Table 1 and Table 2), 13 of which were published in the last decade. Fifteen of the included studies evaluated perioperative antibiotic prophylaxis (PAP) for surgically treated maxillofacial fractures [4,24,25,26,27,28,29,30,31,32,33,34,35,36,37] and one study evaluated systemic antibiotic prophylaxis for conservatively treated fractures [38].

Upper and lower facial fractures were discussed separately because significantly more infections are seen in fractures of the tooth-bearing area of the mandible, which is frequently argued to be due to the proximity to the oral cavity [39]. Table 1 provides an overview of the 11 included studies on lower facial fractures (mandibular fractures) [24,25,26,27,28,29,30,31,32,34,37], and Table 2 provides an overview of the ten studies on midface and upper facial fractures [4,25,26,27,31,33,35,36,37,38]. Five studies included both mandibular as well as midface and upper facial fractures; these results were included in both tables [25,26,27,31,37]. Since the exact type of antibiotic therapy, in relation to the outcome, was not described in most studies, a separate column was provided for the generic names of the administered antibiotics.

In total, we included ten randomized controlled trials (RCT) [4,24,25,26,27,30,32,34,35,36] and six retrospective cohort studies (RCS) [28,29,31,33,37,38], with a total of 2430 patients. For fractures of the lower facial third (Table 1), six studies reported on both open and closed mandibular fractures [24,27,28,29,30,37] and the remaining five studies failed to report if the fractures were open or closed [25,26,31,32,34]. For fractures of the midface and upper facial third (Table 2), two studies only included patients with closed fractures [4,38], one reported on both open and closed fractures [37], and seven studies failed to report if the fractures were open or closed [25,26,27,31,33,35,36].

### 3.1. Study Design and Research Aims

Included studies showed a considerable variability in their design and research aims, which rendered them difficult to compare. Chole et al. compared antibiotic prophylaxis of one hour preoperatively and eight hours postoperatively, to no antibiotic prophylaxis at all [27]. Campos et al. evaluated prolonging antibiotic prophylaxis until 24 h postoperatively to a single-dose administration at the time of induction (20 min before surgery) [26]. Eight studies compared either less than one day (no [25,30], a single-dose [32] or 12 h [24]) or up to 24 h of postoperative antibiotic prophylaxis [31,34,35,36]), to five or more days of postoperative antibiotic prophylaxis [24,25,30,31,32,34,35,36]. Moreover, six studies were included that presented different study designs. Jang et al. compared four days of postoperative antibiotic prophylaxis to one dose at induction [4]. Lovato et al. and Zosa et al. grouped all patients who received antibiotic prophylaxis for less than 24 h and compared them to patients receiving more than 24 h of antibiotic prophylaxis [29,37]. Finally, Domingo et al., Reiss et al., and Malekpour et al. compared antibiotic prophylaxis for variable durations [28,33,38].

### 3.2. Study Outcomes

Infection rates varied from 0% to 43.9% for all included studies in this review [4,24,25,26,27,28,29,30,31,32,33,34,35,36,37,38].

#### 3.2.1. Fractures of the Lower Facial Third

Infection rates for mandibular fractures varied from 3.3% to 43.9% (Table 1). None of the included studies found a statistically significant benefit of prolonging antibiotic prophylaxis over 24 h [24,25,28,29,30,31,32,34,37]. Campos et al. illustrated a significant benefit of continuing antibiotic prophylaxis until 24 h postoperatively [26]; infection rates were almost seven times higher for patients who received only a single dose at the time of induction. Chole et al. could only establish a significant benefit of antibiotic prophylaxis (one hour preoperatively and eight hours postoperatively) for fractures of the mandibular angle or parasymphyseal region treated with open reduction and internal fixation (ORIF) [27]. However, for condylar fractures and for all mandibular fractures treated with closed reduction (CR), no significant reduction was seen in infection rates for patients receiving antibiotic prophylaxis compared to no antibiotic prophylaxis at all [27]. Furthermore, Zosa et al. noted a significantly increased infection rate for patients who received antibiotic prophylaxis over 24 h [37], and Miles et al. described an increased late-onset infection in patients who received antibiotic prophylaxis for five to seven days [30].

#### 3.2.2. Fractures of the Midface and Upper Facial Third

Infection rates for fractures of the midface and upper facial third varied from 0% to 12.5% (Table 2). None of the included studies found a significant benefit in prolonging antibiotic prophylaxis over 24 h [4,25,31,33,35,36,37]. Furthermore, Campos et al. even suggested that a single-dose administration at the time of induction (20 min before surgery) suffices and found no significant benefit of prolonging antibiotic prophylaxis for 24 h [26]. Finally, two studies on surgically treated fractures [27,33] and one study on conservatively treated fractures [38] reported an infection rate of 0% without antibiotic prophylaxis.

#### 3.2.3. Antibiotic Type

All included studies administered β-lactam antibiotics. Nine studies used cephalosporins, including first (cefazolin, cefalexin, cefadroxil, and cefazedone), second (cefotetan), third (ceftriaxone, cefotaxime, and cefdinir), and fourth (cefepime) generation cephalosporins [4,25,26,27,28,29,30,33,37]. Twelve studies used penicillins, including amoxicillin—often co-administered with clavulanic acid or sulbactam—and penicillin G or penicillin VK [24,28,29,30,31,32,33,34,35,36,37,38]. In three studies, the penicillins or cephalosporins were combined with metronidazole [25,30,32]. Clindamycin was the antibiotic of choice in case of a penicillin allergy [29,30,31,38].

Furthermore, four retrospective cohort studies used a wide variety of antibiotics or combinations, without specifying the reasoning for administering different antibiotic types to different patients [28,29,33,37]. Next to cephalosporins and penicillins, fluoroquinolones (moxifloxacin, levofloxacin, and ciprofloxacin), carbapenems (imipenem), cotrimoxazole (sulfamethoxazole and trimethoprim), tetracyclines (doxycycline), and vancomycin were administered [28,29,33,37].

Since none of the included studies compared the different types of antibiotics, and data on pathogens identified by culture at the time of the diagnosis of infection were lacking across studies, no statement could be made about the most suitable antibiotic type used for antibiotic prophylaxis in maxillofacial fracture treatment.

### 3.3. Outcome Description

Infection was the primary outcome measure in all included studies. Lovato et al. and Reiss et al. failed to describe the applied criteria for infection [29,33]. For the included studies that did describe their outcome parameters, there was considerable variation in the definition of infection (Table 3). Six studies referred to the criteria for surgical site infection (SSI) as described by the Centers for Disease Control and Prevention (CDC) [28,31,32,34,35,36]. The remaining studies formulated their own criteria without providing a reference or substantiating the reasoning for the stated criteria [4,24,25,26,27,30,32,38]. Miles et al. [30] described local clinical and radiological criteria for infection, which were subsequently adopted by Perepa et al. [32]. Abubaker et al. [24] described local and systemic clinical criteria, which were subsequently adopted by Baliga et al. and Campos et al. [25,26].

### 3.4. Follow-Up Period

Follow-up periods varied widely and ranged from less than one week to six months, with a median follow-up of six weeks [4,24,25,26,27,28,29,30,31,32,33,34,35,36,37,38]. Two studies (one RCT and one RCS) provided a follow-up of less than one month [25,38]. Reiss et al. included patients with a variable follow-up of less than one month to more than three months [33]. Three more studies (two RCTs and one RCS) provided a follow-up of just one month for all patients [4,27,28]. The RCT by Miles et al. had a follow-up of five weeks [30]. The RCS by Lovato et al. and the RCTs by Campos et al. and Abubaker et al. had a follow-up of six weeks [24,26,29]. Most patients included in the RCS by Zosa et al. were followed for a variable duration of one to eight months [37]. The RCT by Perepa et al. had a follow-up of three months [32]. Only four studies (two RCTs and two RCS) provided a follow-up of six months [31,34,35,36].

## 4. Discussion

This scoping review included ten RCTs and six RCS investigating the effect of systemic antibiotic prophylaxis duration on the infection rate after treatment of maxillofacial fractures [4,24,25,26,27,28,29,30,31,32,33,34,35,36,37,38].

### 4.1. Duration of Systemic Antibiotic Prophylaxis

Based on the included studies, prolonged antibiotic prophylaxis does not appear to be beneficial in the prevention of infection. None of the studies comparing an antibiotic prophylaxis duration of up to 24 h with longer durations found a significant difference in infection rates [4,24,25,28,29,30,31,32,33,34,35,36,37,38].

The results of this scoping review are consistent with the most recent recommendations, which support discontinuing antibiotic prophylaxis after 24 h for all fracture types [10]. For mandibular fractures, continuing antibiotic prophylaxis for 24 h after wound closure is advised, but not beyond this time frame [24,25,26,28,29,30,31,32,34,37]. For upper and midface fractures, the benefit of continuing antibiotic prophylaxis for 24 h is questioned [26].

For conservatively treated fractures of the middle and upper facial third, there is no evidence for the use of systemic antibiotic prophylaxis at all [38].

### 4.2. Clinical Heterogeneity and the Limited Number of High-Quality Studies

Our initial literature search revealed 29 studies on the type and duration of systemic antibiotic prophylaxis with infection as the primary outcome measure [4,24,25,26,27,28,29,30,31,32,33,34,35,36,37,38,39,40,41,42,43,44,45,46,47,48,49,50,51] (Figure 1). Compared to previous systematic reviews, we excluded multiple studies due to the fact that their primary aim was not to investigate the type and duration of systemic antibiotic prophylaxis, due to a lack of comparison group, or due to unclear reporting of the antibiotic prophylaxis duration [39,40,41,42,43,44,45,46,47,48,49,50,52,53,54,55,56,57,58]. One more study was excluded because it unclearly described which fractures were treated surgically and which were treated non-surgically [51]. Previously published systematic reviews included these studies [1,3,7,12,13,14,16,17] and even performed a meta-analysis [1,7,14]. In our opinion, the strict inclusion criteria enhanced the strength of our study and led to a more appropriate representation of available evidence. However, even for the included studies, variability in study design, antibiotic type, surgical treatment, fracture type and location, follow-up period and outcome caused significant heterogeneity.

#### 4.2.1. Patient Characteristics

Eleven of the studies in this scoping review included patients under the age of 18 years [25,26,27,28,29,30,31,33,34,35,36]. The youngest patient, included by Domingo et al., was only 2 years old [28]. Moreover, two studies failed to report the age of the included patients [4,32]. Skeletally immature patients have a superior ability for soft-tissue recovery and infection resistance [59]. As the inclusion of these patients can potentially lead to an underestimation of the actual number of infectious complications, future trials should exclude skeletally immature patients.

#### 4.2.2. Antibiotic Type and Duration

Although all studies administered β-lactam antibiotics, the antibiotic agents that were prescribed varied widely. In retrospective studies, a wide variety of antibiotics or combinations was often administered without specifying the reasoning for administering the different antibiotic types to different patients [28,29,33,37]. Future trials should be based on a single regimen to avoid possible confounding.

Furthermore, the duration of antibiotic prophylaxis varied and none of the studies had the same research question. Of the included prospective studies, one administered antibiotic prophylaxis one hour preoperatively and eight hours postoperatively [27], one evaluated prolonging antibiotic prophylaxis until 24 h postoperatively [26], and ten administered antibiotic prophylaxis for four days [4] or five or more days postoperatively [24,25,29,30,31,32,34,35,36]. Comparison groups received either no dose [4,25,27,29,30], a single dose [26,32], 12 h [24] or up to 24 h [31,34,35,36] of antibiotic prophylaxis. In addition, both treatment arms often received variable preoperative and intraoperative antibiotic prophylaxis, which possibly confounded the study results. Of the included retrospective studies, Domingo et al. and Reiss et al. reported variable and unknown postoperative durations, while patients received variable or no preoperative antibiotics [28,33]. Zosa et al. and Lovato et al. attempted to solve this heterogeneity by grouping all patients receiving antibiotic prophylaxis for less than 24 h and patients receiving antibiotic prophylaxis for more than 24 h [29,37]. Malekpour et al. compared non-surgically treated patients who did not receive antibiotic prophylaxis to those receiving either 1 to 5 days, or more than 5 days of antibiotic prophylaxis [38]. Only Mottini et al. prescribed the same type of antibiotic to all patients (except for patients with a penicillin allergy) and was able to provide a delineated comparison group [31].

#### 4.2.3. Causal Pathogens

The selection of prophylactic antibiotics for infection prevention should consider the susceptibility of potential pathogens to these antibiotics [60]. Therefore, knowledge of the type of causative pathogens in infection related to the treatment of maxillofacial trauma is required. When the source of pathogens is skin flora, cefazolin is the antibiotic of choice as it covers Gram-positive cocci (i.e., *Staphylococcal species*) [60]. In maxillofacial trauma surgery, where pathogens may include oropharyngeal flora (*Streptococcal species*; oropharyngeal anaerobes (i.e., *Peptostreptococcus species*)), broad-spectrum antibiotics may be indicated (amoxicillin with clavulanic acid, or cephazolin with metronidazole) [61]. Data on pathogens identified by culture at the time of diagnosis of infection are lacking in the currently available literature.

The gold standard for a diagnosis of infection remains the deep tissue cultures, obtained from intraoperative samples [62]. Data on other techniques, such as a culture of sonication fluid from hardware, polymerase chain reaction (PCR), and histopathology (i.e., the presence of polymorphonuclear neutrophils (PMNs)) have not been described with respect to the diagnosis of infection after a maxillofacial trauma. For long-bone fractures, sonication of the osteosynthesis material and subsequent inoculation of sonication fluid has already proven to be useful in diagnosing infection [62]. Using low-intensity ultrasound, sonication is deployed to dislodge the biofilm from the osteosynthesis material. The sonication fluid is then cultured onto bacterial media for further analysis [62]. Sonication may be a way to avoid the contamination of tissue cultures with oral flora, and high-quality studies are needed to substantiate the utility of sonication in the treatment of infection after maxillofacial fractures.

Included studies showed considerable variability in their antibiotic type, with varying specificity against the presumed bacterial flora. This is a significant limitation, as the use of an effective antibiotic agent is essential to validate study outcomes. Abubaker et al. administered penicillin G and penicillin VK—narrow-spectrum antibiotics that only cover *Streptococcal species*—which could be a possible cause of their insignificant study results [24]. Furthermore, four retrospective studies administered a wide variety of antibiotic regimens in the intervention arm, resulting in insufficient sample sizes and therefore conclusions that should be interpreted with caution [28,29,33,37].

Future trials should thoroughly describe the causal pathogens related to infection after maxillofacial trauma, identified by culture. High quality, uncontaminated, deep tissue and implant samples are essential to validate culture outcomes [63]. Furthermore, to avoid false-negative culture results, it is generally advised to stop antimicrobial therapy two weeks before sampling [63].

#### 4.2.4. Fracture Type

Five included studies on mandibular fractures failed to report whether they included open or closed fractures [25,26,31,32,34]. A mandibular fracture is considered open when the fracture site communicates either intraorally through the mucosa or extraorally through a laceration or avulsive injury of the overlying skin. Therefore, all fractures involving the tooth-bearing areas of the jaws are regarded as open fractures [64]. Any mandibular fracture that does not have extraoral communication and/or involves the tooth-bearing area is considered a closed fracture (e.g., condylar fracture) [64]. Numerous studies have shown that an open fracture is considered a significant risk for infection [27,28,39]. Therefore, not reporting whether the included fractures are open or closed makes the interpretation of the study results almost impossible. As most open fractures are contaminated with microorganisms, immediate antibiotic administration, wound debridement, soft-tissue coverage, and fracture stabilization are necessary [65]. While an immediate antibiotic administration at admission is the standard of care for long-bone open fractures [65], evidence is lacking for maxillofacial fractures and studies have not been able to objectify this benefit [47], possibly due to the limited number of included patients and insufficient follow-up. There is a need for high-quality studies evaluating the benefit of starting antibiotic prophylaxis at admission for open maxillofacial fractures.

#### 4.2.5. Surgical Treatment

Fracture stability is of the utmost importance in the prevention of infection. Instability leads to ongoing soft-tissue trauma, interruption of neo-vascularity and osteolysis of bone, which creates a supportive environment for bacterial proliferation [66]. However, in most studies included in this review, both surgical therapy (e.g., fracture stability) and the timing of fracture fixation were poorly described [24,26,27,28,29,30,31,32,33,37,38].

Surgical treatment of mandibular fractures is performed using ORIF or CR and maxillomandibular fixation (MMF). Standard MMF methods are either tooth-supported (arch bars, interdental wires, or Ernst ligatures), or bone-supported devices such as intermaxillary fixation (IMF) screws [64]. Out of 11 included studies on surgically treated mandibular fractures, only Chole et al. and Domino et al. report separate infection rates for CR-MMF and ORIF [27,28]. These data are important as an open procedure may lead to a four-fold higher rate of infection [28,67]. As the type and timing of fracture fixation can have an influence on the outcome, future trials should develop protocols that clearly take these aspects into account.

Only one study on non-surgically treated maxillofacial fractures met our inclusion criteria [38]. The study by Malekpour et al. included 289 patients with maxillary and orbital fractures. They compared no antibiotic prophylaxis to 1 to 5 days, or more than 5 days of antibiotic prophylaxis, and showed an infection rate of 0% for all three comparison groups [38]. Based on the available literature, there is no evidence of the utility of systemic antibiotic prophylaxis in conservatively treated maxillofacial fractures [38,40,51,55,56]. However, due to the considerable risk of bias, it is not possible to withhold guidelines based on these studies. High-quality studies are needed to clarify the role of antibiotic use with respect to infection prevention and treatment in conservatively treated maxillofacial fractures.

#### 4.2.6. Outcome Description

The lack of a uniform definition for infection after maxillofacial trauma also contributed to the scarcity of comparable data. Using inadequate outcome parameters risks underestimating or overestimating the actual number of complications, resulting in misleading study conclusions [5]. The absence of a universally accepted definition of infection after maxillofacial trauma mirrors the situation for fracture-related infection (FRI) in long-bone fractures and prosthetic joint infection (PJI) identified many years ago [68].

To date, the term FRI has not been used to describe infection following maxillofacial trauma. Although general treatment principles may differ, and significant differences exist with respect to the blood supply at the fracture site and the bacterial flora present at the site of injury, the basic diagnostic principles are similar. The definition of FRI is based on clinical, laboratory and radiological features that confirm or exclude the presence of infection. The described confirmatory and suggestive criteria could also apply to maxillofacial trauma and possibly be utilized to diagnose (and define) infection [5]. The term FRI covers both surgically treated as well as conservatively treated fractures, which is why we prefer to use the standardized term of FRI even though it has not yet been widely accepted within oral and maxillofacial surgery practice.

#### 4.2.7. Follow-Up Period

In this review, follow-up periods were mostly short with only four studies reaching six months [31,34,35,36]. This suggests that late-onset infections were probably missed in most studies. The CDC currently advocates a surveillance period of 90 days after fracture fixation [69]. In clinical studies on maxillofacial trauma, a follow-up over 90 days is rare. A recent retrospective study on the timing of infection onset in patients with long-bone fractures illustrated that in a follow-up period of 90 days, only 64% of infections were diagnosed, while after one year of follow-up this percentage increased to 89% [70]. Of course, we should be careful with extrapolating these results to patients with maxillofacial fractures. Since failure to diagnose late-onset infections may lead to misrepresentation of study results, a minimum follow-up period of one year is recommended to ensure a correct representation of the study outcome [70].

### 4.3. Discrepancy between Guidelines and Clinical Practice

To the best of our knowledge, 11 systematic reviews on systemic antibiotic prophylaxis in maxillofacial fracture treatment were published over the past 15 years [1,3,6,7,11,12,13,14,15,16,17]. The first systematic review by Andreasen et al., in 2006, already demonstrated that 24 h of antibiotic prophylaxis suffices as antimicrobial prophylaxis in all maxillofacial fractures [6]. These findings were confirmed in all subsequent systematic reviews [1,3,7,11,12,13,14,15,16,17].

Recent surveys have confirmed that surgeons do not always adhere to these recommendations, and a high percentage still prolong the use of antibiotic prophylaxis [16,17]. A survey on systemic antibiotic prophylaxis in patients undergoing ORIF for mandibular fractures, where 687 surgeons responded, showed that 75% of surgeons stated that they administer antibiotics for up to 3 days (44.1%), 1 week (54.8%), and more than 1 week (1.1%) in the case of open fractures. Furthermore, 51% of surgeons administer antibiotics for up to 3 days (50.5%), 1 week (48.6%), and more than 1 week (1%) in the case of closed fractures [17].

The question arises as to why the above-mentioned recommendations have not yet been implemented in our daily clinical practice. Existing guidelines are mainly based on only a handful of clinical studies which still leads to uncertainty regarding the optimal type and duration of systemic antibiotic prophylaxis. Therefore, worldwide heterogeneity in prevention protocols still exists. Because of the microbiome present in the oral cavity and sinuses, maxillofacial surgeons fear contamination of the fracture site with commensal flora leading to infection [17] and intuitively assume that there may be a benefit to prolonged antibiotic administration. Since high-quality data for procedures that involve clean-contaminated procedures are lacking, controversy related to this topic remains within the maxillofacial community. There is a need for large, multicentric, well designed, double-blind RCTs, with sufficient follow-up, that can contribute to the guidelines with respect to the diagnosis and treatment of FRI in maxillofacial trauma.

### 4.4. Implications of Antibiotic Overuse

Antibiotics are a worldwide leading cause of adverse drug reactions and emergency department visits [71]. Prolonged systemic antibiotic prophylaxis can be associated with, rash, diarrhea and *Clostridioides difficile* infection, nausea, vomiting and abdominal pain, malaise, and fatigue [72,73].

Furthermore, prolonged antibiotic prophylaxis could possibly contribute to delayed infectious complications. Zosa et al. described a significantly increased infection risk for patients receiving prolonged antibiotic prophylaxis over seven days [37], and Miles et al. found that patients who received extended antibiotic prophylaxis developed late infectious complications [30]. A recent systematic review by Delaplain et al. confirmed a higher rate of surgical site infection for more than 72 h of antibiotic prophylaxis [7]. This issue has also been an issue encountered in long-bone fractures [74]. To the best of our knowledge, a possible cause for this phenomenon has not yet been described in the current literature and further research is required to validate these findings.

Finally, the overuse and improper use of antibiotics are considered important drivers for the emergence and spread of AMR [75]. AMR occurs as a natural evolutionary response to antimicrobial exposure, whereby microorganisms acquire the ability to withstand antimicrobial drugs via mutations in chromosomal genes and by horizontal gene transfer [75]. The global spread of AMR may compromise our ability to treat existing and emerging common infectious diseases, as well as undermining many other improvements in health care. Maxillofacial surgeons, like all healthcare workers, should realize that AMR is a global health problem. Antimicrobial stewardship promotes the appropriate use of antibiotics based on internationally accepted guidelines, leading to effective prevention and treatment of infections while avoiding the harmful effects of antibiotic use [75].

## 5. Limitations

The main limitation of this scoping review is the limited quality and heterogeneity of evidence available in the literature. In our opinion, none of the current studies provides sufficient evidence to serve as a starting point for future RCTs. Variability in study design, antibiotic type and duration, treatment, fracture type and location, renders existing studies difficult to evaluate and compare. Outcome measurements were mostly determined using unclear criteria and inadequate follow-up periods. Therefore, this review only provides limited evidence, and the results should be interpreted with caution.

## 6. Conclusions

This scoping review demonstrates the lack of well-constructed studies investigating the association between the type and duration of systemic antibiotic prophylaxis and infection in the treatment of maxillofacial fractures. All current available studies with respect to this topic showed a significant risk of bias. The considerable variability in antibiotic duration seen throughout the literature highlights the need for further research and guidelines for the use of antibiotic prophylaxis in maxillofacial trauma. Based on currently available evidence, shortening the duration of antibiotic prophylaxis to one day or less for all operatively treated facial fractures should be sufficient. Furthermore, there is no evidence for the use of systemic antibiotic prophylaxis in conservatively treated fractures. In fact, overprescribing practices may contribute to increased long-term infectious complications and AMR. Furthermore, although multiple guidelines have been developed to prevent infections after maxillofacial trauma over recent years, worldwide heterogeneity still exists with respect to the implementation of these guidelines in our daily clinical practice. High-quality studies are needed to clarify the optimal duration of antibiotic prophylaxis in the management of maxillofacial fractures.

## Figures and Tables

**Table 1:** Characteristics of included studies: Lower facial third (mandibular) fractures.

Author	Risk of Bias *	Open or Closed Fractures	Treatment	N	Antibiotic Type	Comparison *Antibiotic Regimen for All Patients*	Outcome (IR = Infection Incidence Rate)	Follow-Up
**Randomized controlled trials**								
*Surgically treated fractures*								
**Campos** **2015** **(Part I)**	High risk of bias	NR	Surgical (NS)	31	cefazolin	Postoperative Ab every 6 h for 24 h vs. no postoperative Ab *All patients received Ab 20 min before surgery.*	IR: 24 h postoperative Ab 5.6%, no postoperative Ab 38.5% *Significant difference between the regimens.*	6 w
**Chole** **1987** **(Part I)**	High risk of bias	Open and closed	ORIF and CR-MMF	79	cefazolin	Ab 1 h preoperatively and 8 h later vs. no Ab	IR: Ab 13.2 %, no Ab 43.9% *Significant difference between the regimens.*	30 d
**Baliga** **2014** **(Part I)**	High risk of bias	NR	ORIF	30	cefotaxime, metronidazole	5 d postoperative Ab vs. no postoperative Ab *All patients received Ab pre- and intra-operatively*	IR: Ab group 3.3%; no Ab group 3.3% *No significant difference between the regimens.*	3 w
**Abubaker 2001**	High risk of bias	Open and closed	ORIF and CR-MMF	30	penicillin G and penicillin VK	5 d postoperative Ab vs. no prolonged postoperative Ab *All patients received Ab every 4 h preoperatively, intraoperatively and 12 h postoperatively*	IR: 5 d postoperative Ab 14.3%, no prolonged postoperative Ab 12.5% *No significant difference between the regimens.*	6 w
**Perepa** **2018**	High risk of bias	NR	ORIF	144	amoxicillin/ clavulanic acid, metronidazole	5 d postoperative Ab vs. no prolonged postoperative Ab *All patients received preoperative Ab and 1 dose iv postoperative*	IR: 5 d postoperative Ab 20.5%, no prolonged postoperative Ab 20% *No significant difference between the regimens.*	3 m
**Schaller** **2013**	High risk of bias	NR	ORIF	59	amoxicillin/ clavulanic acid	5 d postoperative Ab vs. no prolonged postoperative Ab *All patients received Ab from admission until 24 h postoperatively*	IR: 5 d postoperative Ab 20%, no prolonged postoperative Ab 21% *No significant difference between the regimens.*	6 m
**Miles** **2006**	High risk of bias	Open and closed	ORIF	181	penicillin G, metronidazole, cephalosporins, cefazolin, clindamycin	5–7 d postoperative Ab vs. no postoperative Ab *All patients received Ab from diagnosis until surgery and intraoperatively*	IR: 5–7 d postoperative Ab 9.9%, no postoperative Ab 14.0% *No significant difference between the regimens.*	5 w
**Retrospective cohort studies**								
*Surgically treated fractures*								
**Lovato** **2009**	Serious risk of bias	Open and closed	ORIF and CR-MMF	150	cefazolin, cefalexin, cefepime, cefotetan, clindamycin, doxycycline, imipenem, penicillin, amoxicillin/clavulanic acid, amoxicillin/sulbactam, piperacillin/tazobactam, unknown (18%)	≤24 h postoperative Ab vs. >24 h postoperative Ab (24 h–10 d) *All patients received perioperative Ab for* ≤24 h	IR: <24 h postoperative Ab 13.33%, >24 h postoperative Ab 10.67% *No significant difference between the regimens.*	6 w
**Mottini** **2014** **(Part I)**	Serious risk of bias	NR	NR	115	amoxicillin/ clavulanic acid or clindamycin	≥5 d postoperative Ab vs. no prolonged postoperative Ab *All patients received Ab from admission until 24 h postoperatively.*	IR: ≥5 d postoperative Ab 9.59%, no prolonged postoperative Ab 11.90% *No significant difference between the regimens.*	6 m
**Domingo 2016**	Critical risk of bias	Open and closed	ORIF and CR-MMF	359	cefazolin, cefalexin, cefadroxil, cefepime ceftriaxone, amoxicillin, amoxicillin/clavulanic acid, amoxicillin/sulbactam, moxifloxacin, levofloxacin, ciprofloxacin, bactrim, clindamycin, combinations	Postoperative Ab (1–3 d, 4–7 d, >7 d, or unknown) vs. no postoperative Ab *Patients received variable or no preoperative Ab.*	IR: postoperative Ab 14.7%, no postoperative Ab 9.6% *No significant difference between the regimens.*	4 w
**Zosa** **2021** **(Part I)**	Serious risk of bias	Open and closed	NR	42	First- generation cephalosporins, β-lactam antibiotics with β-lactamase inhibitors, clindamycin, penicillin, vancomycin, tetracycline, and combinations.	<24 h Ab (incl. single-dose or no Ab) vs. >24 h Ab (median 4 d (range 1–14 d))	IR: <24 h Ab 4.0%, >24 h Ab 29.4% *Significantly higher infection rates for longer courses.*	1–30 m (8 m) (Follow-up rate 93.6%)

Abbreviations: N: number of patients; IR: infection incidence rate; RCT: randomized controlled trial; RCS: retrospective cohort study; NR: not reported; NS: not specified; ORIF: open reduction and internal fixation; CR-MMF: closed reduction–maxillomandibular fixation; Ab: antibiotics; min: minutes, h: hours, w: weeks; m: months; incl.: including. * As evaluated by the Risk Of Bias In Non-Randomized Studies assessment tool (ROBINS-I) [22], or the revised Cochrane Risk-of-Bias tool for randomized trials (RoB 2) [23].

**Table 2:** Characteristics of included studies: Middle and upper facial third fractures.

Author	Risk of Bias *	Fracture Location	Open or Closed Fractures	N	Antibiotic Type	Comparison *Antibiotic Regimen for All Patients*	Outcome (IR = Infection Incidence Rate)	Follow-Up
**Randomized controlled trials**								
*Surgically treated fractures*								
**Campos** **2015** **(Part II)**	High risk of bias	Midface, upper face	NR	44	cefazolin	Postoperative Ab every 6 h for 24 h vs. no postoperative Ab *All patients received Ab 20 min before surgery.*	IR: 24 h postoperative Ab 0%, no postoperative Ab Ab 3.3% *No significant difference between the regimens*	6 w
**Chole** **1987** **(Part II)**	High risk of bias	Zygoma, Le Fort	NR	101	cefazolin	1 h preoperative and 8 h later vs. no Ab	IR: 0% *No significant difference between the regimens.*	30 d
**Baliga** **2014** **(Part II)**	High risk of bias	Zygoma	NR	30	cefotaxime, metronidazole	5 d postoperative Ab vs. no postoperative Ab *All patients received Ab pre- and intra-operatively*	IR: 0.00% *No significant difference between the regimens.*	3 w
**Jang** **2019**	High risk of bias	Nasal bone	Closed	30	cefazedone, cephalexin	Ab 4 d postoperatively vs. no postoperative Ab *All patients received one dose of Ab at induction.*	IR: 0% *No significant difference between the regimens.*	30 d
**Zix** **2013**	High risk of bias	Orbit	NR	62	amoxicillin/ clavulanic acid	5 d postoperative Ab vs. no prolonged postoperative Ab *All patients received Ab from admission until 24 h postoperatively.*	IR: 5 d postoperative Ab 6.8%, no prolonged postoperative Ab 3.2% *No significant difference between the regimens.*	6 m
**Soong** **2014**	High risk of bias	Le Fort, zygoma	NR	94	amoxicillin/ clavulanic acid	5 d postoperative Ab vs. no prolonged postoperative Ab *All patients received Ab from admission until 24 h postoperatively.*	IR: 5 d postoperative Ab 4.4%, no prolonged postoperative Ab 4.1% *No significant difference between the regimens.*	6 m
**Retrospective cohort studies**								
*Surgically treated fractures*								
**Reiss** **2017**	High risk of bias	Orbit	NR	172	cefazolin, cephalexin, cefdinir, ceftriaxone, penicillin, amoxicillin, amoxicillin/clavulante, ampicillin/sulbactam, piperacillin/tazobactam, clindamycin ciprofloxacin, levofloxacin, azithromycin, vancomycin	No Ab, vs. one dose of Ab, vs. 5–7 days of Ab, vs. 10–14 d of Ab	IR: 0.00% *No significant difference between the regimens.*	<1 w–>3 m
**Mottini** **2014** **(Part II)**	Serious risk of bias	Zygoma, orbit, Le Fort	NR	339	amoxicillin/ clavulanic acid or clindamycin	≥5 d postoperative Ab vs. no prolonged postoperative Ab *All patients received Ab from admission until 24 h postoperatively.*	IR: ≥5 d postoperative Ab 0.4%, no prolonged postoperative Ab 0%, *No significant difference between the regimens.*	6 m
**Zosa** **2021** **(Part II)**	Serious risk of bias	Midface	Open and closed	49	First- generation cephalosporins, β-lactam antibiotics with β-lactamase inhibitors, clindamycin, penicillin, vancomycin, tetracycline, and combinations.	<24 h Ab (incl. single-dose or no Ab) vs. >24 h Ab (median 4 d (range 1–14 d))	IR: <24 h Ab 7.3%, >24 h Ab 12.5% *No significant difference between the regimens.*	1–30 m (8 m) (Follow-up rate 93.6%)
*Conservatively (non-surgically) treated fractures*								
**Malekpour** **2016**	High risk of bias	Maxilla, orbit	Closed	289	ampicillin/sulbactam, amoxicillin/clavulanate, or combinations, clindamycin	No Ab, vs. 1–5 d of Ab, vs. >5 days of Ab	IR: 0.00% *No significant difference between the regimens.*	2 w

Abbreviations: N: number of patients; IR: infection incidence rate; RCT: randomized controlled trial; RCS: retrospective cohort study; NR: not reported; NS: not specified; Ab: antibiotics; min: minutes, h: hours, w: weeks; m: months; incl.: including. * As evaluated by the Risk Of Bias In Non-Randomized Studies assessment tool (ROBINS-I) [22], or the revised Cochrane Risk-of-Bias tool for randomized trials (RoB 2) [23].

**Table 3:** Outcome description: definition of infection.

Author	Outcome Description: Definition of Infection
**Lovato 2009** **Reiss 2017**	Not reported
**Domingo 2016** **Perepa 2018** **Schaller 2013** **Mottini 2014** **Soong 2014** **Zix 2013**	CDC guidelines
**Miles 2006** **Perepa 2018**	Clinical criteria: Grade I: Erythema around suture line < 1 cm; Grade II: 1–5 cm of erythema; Grade III: > 5 cm of erythema and induration; Grade IV: Purulent drainage spontaneously or by incision; Grade V: Fistulae Radiological criteria: Grade I: Ossification of fracture site/no change from initial injury; Grade II: Radiolucenties localized to hardware or necrotic tooth; Grade III: Generalized radiolucenties of fracture or hardware
**Abubaker 2001** **Baliga 2014** **Campos 2015**	Purulent drainage from the surgical or fracture site, increased facial Swelling beyond postoperative day 7, fistula formation at the surgical or fracture site, with evidence of drainage, fever associated with local evidence of infection (swelling, erythema, or tenderness).
**Jang 2019**	Nasal bone infection: Heating sensation, swelling, persistent pain, purulent nasal drainage, septal abscess, vital sign showing general signs of infection (not specified).
**Malekpour 2016**	Warmth, redness, abscess, fever, purulent drainage, or patients who were started on antibiotics at follow-up.
**Chole 1987**	The fracture site, incision, or adjacent area showed clinical signs of infection, including purulent drainage, abscess formation, or cellulitis.

## Supplementary Materials

The following supporting information can be downloaded at: https://www.mdpi.com/article/10.3390/antibiotics11040483/s1. Search strings for each database are provided as Supplementary Data.
