# Supplementary material for: Systemic Antibiotic Prophylaxis in Maxillofacial Trauma: A Scoping Review and Critical Appraisal
Source: Antibiotics (Basel). 2022 Apr 5;11(4):483. doi: 10.3390/antibiotics11040483 (PMC9027173; doi:10.3390/antibiotics11040483)
Supplement: Supplementary file 1 [file antibiotics-11-00483-s001.zip › antibiotics-1625233-supplementary.pdf]

Supplementary data: Search strings for each database.

1. Pubmed (Medline)

Concept 1: Maxillofacial Trauma

"Maxillofacial Injuries"[Mesh] OR "Skull Fracture, Basilar"[Mesh] OR (("injur*"[tiab] OR "trauma*"[tiab] OR "fracture*"[tiab]) AND ("jaw"[tiab] OR "jaws"[tiab] OR "orbit*"[tiab] OR "zygoma*"[tiab] OR "temporal bone*"[tiab] OR "maxillofacial*"[tiab] OR "maxillo facial*"[tiab] OR "facial"[tiab] OR "face"[tiab] OR "mandible*"[tiab] OR "mandibular*"[tiab] OR "maxilla*"[tiab] OR "Le Fort*"[tiab] OR "LeFort*"[tiab] OR "midface"[tiab] OR "mid face"[tiab] OR "midfacial"[tiab] OR "craniofacial"[tiab] OR "craniomaxillofacial"[tiab] OR "cheekbone*"[tiab] OR "cheek bone*"[tiab] OR "condylar"[tiab] OR "condyle*"[tiab] OR "skullbase"[tiab] OR "skull base"[tiab] OR "nasal"[tiab] OR "nose"[tiab] OR "frontobasilar"[tiab] OR "frontal bone"[tiab] OR "forehead"[tiab] OR "basilar skull"[tiab] OR "frontobasal"[tiab] OR "panfacial"[tiab] OR "pan facial"[tiab] OR "sinus*"[tiab]))

Concept 2: Antibiotic prophylaxis

"Antibiotic Prophylaxis"[Mesh] OR "Anti-Bacterial Agents"[Mesh] OR "antibiotic*"[tiab] OR "antibacterial*"[tiab] OR "anti bacterial*"[tiab] OR "antiinfect*"[tiab] OR "anti infect*"[tiab] OR (("infect*"[tiab]) AND ("prevent*"[tiab] OR "prophylax*"[tiab]))

2. Embase

Concept 1: Maxillofacial Trauma

'face fracture'/exp OR 'temporal bone fracture'/exp OR 'skull base fracture'/exp OR (('injur*' OR 'trauma*' OR 'fracture*') NEAR/5 ('jaw*' OR 'orbit*' OR 'zygoma*' OR 'maxillofacial*' OR 'maxillo facial*' OR 'facial*' OR 'face*' OR 'mandible*' OR 'mandibular*' OR 'maxilla*' OR 'Le Fort*' OR 'LeFort*' OR 'midface*' OR 'mid face*' OR 'midfacial*' OR 'craniofacial' OR 'cheekbone*' OR 'cheek bone' OR 'condylar' OR 'condyle*' OR 'skullbase' OR 'skull base' OR 'nasal' OR 'nose' OR 'frontobasilar' OR 'frontal bone' OR 'forehead' OR 'basilar skull' OR 'frontobasal' OR 'panfacial' OR 'pan facial' OR ‘sinus*')):ti,ab,kw

Concept 2: Antibiotic prophylaxis

'antibiotic prophylaxis'/exp OR 'antibiotic agent'/exp OR 'antiinfective agent'/exp OR 'infection prevention'/exp OR 'antibiotic*':ab,ti,kw OR 'anti bacterial*':ab,ti,kw OR 'antibacterial':ab,ti,kw OR 'antiinfect*':ab,ti,kw OR 'anti infect*':ab,ti,kw OR (infection NEAR/5 prevent*):ab,ti,kw OR (infection NEAR/5 prophylax*):ab,ti,kw

3. Web of Science Core Collection

Concept 1: Maxillofacial Trauma

TS=(("injur*" OR "trauma*" OR "fracture*") NEAR/5 ("jaw*" OR "orbit*" OR "zygoma*" OR "temporal bone*" OR "maxillofacial*" OR "facial" OR "face" OR "mandible*" OR "mandibular*" OR "maxilla*" OR "Le Fort*" OR "LeFort*" OR "midface" OR "mid face" OR "midfacial" OR "craniofacial" OR "craniomaxillofacial" OR "cheekbone*" OR "cheek bone*" OR "condylar" OR "condyle*" OR "skullbase" OR "skull base" OR "nasal" OR "nose" OR "frontobasilar" OR "frontal bone" OR "forehead" OR "basilar skull" OR "frontobasal" OR "panfacial" OR "pan facial" OR "sinus*"))

Concept 2: Antibiotic prophylaxis

TS=("antibiotic*" OR "antibacterial*" OR "anti bacterial*" OR "antiinfect*" OR "anti infect*" OR (infection NEAR/5 prophylax*) OR (infection NEAR/5 prevent*))

4. Central (Cochrane library)

Concept 1: Maxillofacial Trauma

#1: [mh "Skull Fracture, Basilar"] OR [mh "maxillofacial injuries"] OR ((injur* OR trauma* OR fracture*) NEAR/5 (jaw* OR orbit* OR zygoma* OR (temporal NEXT bone*) OR maxillofacial* OR facial OR face OR mandible* OR mandibular* OR maxilla* OR Le Fort* OR LeFort* OR midface OR mid face OR midfacial OR craniofacial OR craniomaxillofacial OR cheekbone* OR (cheek NEXT bone*) OR condylar OR condyle* OR skullbase OR "skull base" OR nasal OR nose OR frontobasilar OR (frontal NEXT bone) OR forehead OR "basilar skull" OR frontobasal OR panfacial OR "pan facial" OR sinus*)):ab,ti,kw

Concept 2: Antibiotic prophylaxis

#2: [mh "Antibiotic Prophylaxis"] OR [mh "Anti-Bacterial Agents"] OR (antibiotic* OR antibacterial* OR (anti NEXT bacterial*) OR antiinfect* OR (anti NEXT infect*) OR (infection NEAR/5 prophylax*) OR (infection NEAR/5 prevent*)):ab,ti,kw
